# Progression of Chronic Kidney Disease to Dialysis in the Roma Population With Type 2 Diabetes Mellitus in Comparison With Caucasian Patients

**DOI:** 10.7759/cureus.62118

**Published:** 2024-06-10

**Authors:** Andrada Cosoreanu, Emilia Rusu, Florin Rusu, Silviu Stanciu, Georgiana Enache, Gabriela Radulian

**Affiliations:** 1 Diabetes and Endocrinology, “Carol Davila” University of Medicine and Pharmacy, Bucharest, ROU; 2 Diabetes, “Carol Davila” University of Medicine and Pharmacy, Bucharest, ROU; 3 Diabetes, "Nicolae Malaxa" Clinical Hospital, Bucharest, ROU; 4 Urology, “Doctor Carol Davila” Central Military University Emergency Hospital, Bucharest, ROU; 5 Noninvasive Cardiovascular Laboratory, “Carol Davila” University of Medicine and Pharmacy, Bucharest, ROU; 6 Diabetes, “Pompei Samarian” Emergency Hospital, Calarasi, ROU

**Keywords:** roma population, caucasian patients, type 2 diabetes mellitus, risk progression to dialysis, chronic kidney disease

## Abstract

Background

Chronic kidney disease (CKD) poses a significant health challenge among patients, contributing to substantial morbidity and mortality outcomes. However, there remains a paucity of data within the medical literature on the Roma population, one of the most significant minority groups globally, with studies indicating that these individuals are disproportionately affected by CKD compared to the general population, with higher prevalence rates.

Materials and methods

We conducted an observational, cross-sectional study from October 2022 to March 2024, evaluating 735 adult patients with type 2 diabetes mellitus, of which 402 were Roma, aged 18 to 89 years, following the hospital's standard protocols for diabetes management, at the "Nicolae Malaxa" Clinical Hospital in Bucharest, Romania, a tertiary care center for diabetes.

Results

The prevalence of CKD was higher among the Roma patients, reaching 56.50% (n=203), in comparison with the Caucasian group (43.50%, n=156), however, with a lower mean age (55.53±10.56 years versus 63.32±10.04 years). Roma patients with CKD had a higher prevalence of cardiovascular disease compared to Caucasians, including myocardial infarction, stroke, stable angina, and heart failure. Cardiovascular risk factors, such as hypertension, obesity, and dyslipidemia, in patients with CKD, were also more prevalent among the Roma population. Taking into consideration the natural progression of CKD, the anthropometric measurements and laboratory parameters stratified by ethnicity revealed that Roma patients in the very high risk of CKD progression category had a lower mean age, and a lower median duration of diabetes (56.37±10.79 versus 59.92±7.48 years, and 4.00±2.00 versus 10.00±10.30 years, respectively), as well as a more elevated mean waist circumference (WC), body mass index (BMI), total cholesterol (TC), and low-density lipoprotein-cholesterol (LDL-c) compared to Caucasians. Moreover, patients in the very high risk of CKD progression category among both groups showed the highest level of insulin resistance, measured by the triglyceride-glucose (TyG) index (mean value of 10.13±0.60 among the Roma patients, and 10.09±0.82 among Caucasians). Among the study group, weight, WC, BMI, and A Body Shape Index (ABSI) were associated with a very high risk of progression of CKD. In Caucasian patients, it was demonstrated that weight, WC, BMI, ABSI, and triglycerides (TG) have contributed to the very high risk of progression of CKD, while among the Roma patients, no association was found.

Conclusion

In conclusion, our findings suggested a high prevalence of CKD among both groups. There is still a need for further investigation of additional risk factors, such as genetics, limited access to health education, and appropriate treatments that could synergistically accelerate the progression of CKD among Roma patients.

## Introduction

Chronic kidney disease (CKD) poses a significant health challenge among patients, contributing to substantial morbidity and mortality rates, by elevating cardiovascular risk and healthcare expenditures; estimates showed no less than 800 million cases of all-stage CKD globally, with more than half affecting people diagnosed with diabetes mellitus (DM) [1].

According to the American Diabetes Association, in adult patients, CKD affects between 20 and 40% of individuals with diabetes and it may be present at the time of type 2 diabetes mellitus (T2DM) diagnosis [2].

Characterized by the progressive decline in kidney function over time, CKD in diabetes arises primarily due to the damaging effects of prolonged hyperglycemia on the renal microvasculature, although the pathophysiology is multifactorial, involving complex interplays between metabolic, hemodynamic, and inflammatory pathways [1]. As one of the leading causes of end-stage renal disease (ESRD) worldwide, diabetic CKD reflects a considerable burden on healthcare systems and underscores the urgent need for effective prevention and management strategies [1].

In patients with T2DM, a higher body mass index (BMI) is associated with an increased risk of overall diabetic microvascular complications. Apart from this, it is well established that between abdominal obesity and endothelial dysfunction, there is a positive correlation, a higher A Body Shape Index (ABSI) indicating an increased prevalence of cardiovascular risk factors [3,4].

However, there remains a paucity of data within the medical literature on the Roma population, one of the most significant minority groups globally; studies indicate that these individuals are disproportionately affected by CKD compared to the general population, with higher prevalence rates and poorer outcomes, often exacerbated by socio-economic disparities and cultural factors [5,6]. Some of the factors contributing to this disparity include socio-economic disadvantages, such as limited access to healthcare, poor living conditions, and low educational attainment, which can hinder early detection and management of CKD among Roma communities. Additionally, cultural beliefs and practices may influence health-seeking behaviors, leading to delayed diagnosis and treatment initiation [7,8].

Therefore, the aim of this study was to assess the progression of CKD to dialysis in the Roma population with T2DM in comparison with Caucasian patients.

## Materials and methods

Study design and setting

We conducted an observational, cross-sectional study from October 2022 to March 2024, evaluating 735 adult patients with T2DM, of which 402 were Roma, aged 18 to 89 years, following the hospital's standard protocols for diabetes management, at the "Nicolae Malaxa" Clinical Hospital in Bucharest, Romania, a tertiary care center for diabetes. The study adhered to the Strengthening the Reporting of Observational Studies in Epidemiology (STROBE) guidelines [9]. The Ethics Committee for Clinical Studies of the "Nicolae Malaxa" Clinical Hospital approved the study (approval number 75/2022). All participants provided informed consent for data collection and the use of their medical information for research purposes.

Study population

The study included adult patients diagnosed with T2DM who attended consultations at the hospital's inpatient department during the study period and provided informed consent to participate. Conversely, exclusion criteria comprised patients younger than 18 years, those without diabetes, pregnant women, and those who declined to sign the informed consent form.

Data collection

Data collection encompassed ethnicity, age, duration of diabetes, personal medical history of myocardial infarction, stable angina, stroke, heart failure, peripheral artery disease, lower limb amputations, hypertension, obesity, hepatic steatosis, dyslipidemia, metabolic syndrome, presence of microvascular diabetes-related complications (chronic kidney disease, retinopathy, peripheral neuropathy, orthostatic hypotension), anthropometric indicators, and paraclinical assessments.

Clinical measurements

Regarding the clinical measurements, the following anthropometric indicators were assessed for each participant, including height (cm), weight (kg), waist circumference (WC, cm), hip circumference (HC, cm), body mass index (BMI, kg/m^2^) and A Body Shape Index (ABSI).

WC and HC were determined using a measuring tape, following the standard procedures.

The ABSI was calculated using the following formula: WC (m) / [BMI^2/3^ (kg/m^2^) × height^1/2^ (m)] [3].

Paraclinical assessment

Concerning the paraclinical assessment, the laboratory parameters analyzed were fasting plasmatic glycemia (FPG), glycated hemoglobin (HbA1c) level, serum creatinine, estimated glomerular filtration rate (eGFR), urinary albumin-to-creatinine ratio (UACR), uric acid, serum urea, aspartate aminotransferase (AST), alanine aminotransferase (ALT), gamma-glutamyl transferase (GGT), total cholesterol (TC), high-density lipoprotein-cholesterol (HDL-c), low-density lipoprotein- cholesterol (LDL-c), triglycerides (TG), and triglyceride-glucose (TyG) index, a surrogate indicator for the evaluation of insulin resistance.

HbA1c levels were determined using the validated high-performance liquid chromatography (HPLC) method [10]. The eGFR was assessed using the 2021 Chronic Kidney Disease Epidemiology Collaboration (CKD-EPI) formula [11]. The UACR was calculated from spot urine samples by dividing the total urinary albumin value in milligrams by the creatinine concentration in grams.

LDL-c levels were derived either by calculation using the formula: TC minus HDL-c minus TG/5 if TG levels were below 400 mg/dL, or by direct laboratory measurement if TG levels exceeded 400 mg/dL [12]. TyG was calculated using the formula: ln [TG (mg/dL) x FPG (mg/dL)/2] [13].

Definitions

DM was diagnosed in accordance with the criteria outlined by the American Diabetes Association (ADA) guidelines [2]. Resting blood pressure was assessed using the auscultatory method while the patient was seated. Hypertension was defined by systolic blood pressure (SBP) surpassing 140 mmHg, diastolic blood pressure (DBP) exceeding 90 mmHg as per the American Heart Association (AHA) guidelines [14], current use of blood pressure-lowering medication, or documented medical history of physician-diagnosed hypertension.

To categorize nutritional status based on BMI, we adhered to the World Health Organization (WHO) standards: underweight was indicated by a BMI <18.5 kg/m^2^, normal weight by a BMI between 18.5 and 24.9 kg/m^2^, overweight by a BMI between 25 and 29.9 kg/m^2^, and obesity by a BMI exceeding 30 kg/m^2^ [15].

The diagnosis of hepatic steatosis was established through either imaging techniques, such as abdominal ultrasound performed during hospitalization, or a documented medical history of it.

Dyslipidemia diagnosis was based on laboratory tests of the lipid profile in accordance with current guidelines or the patient's current use of lipid-lowering medication [12].

Metabolic syndrome was diagnosed using the harmonized definition, requiring the presence of any three of the following criteria: waist circumference >94 cm for men and >80 cm for women, triglycerides (TG) ≥ 150 mg/dl or specific treatment for this lipid abnormality, low high-density lipoprotein cholesterol (HDL-c) < 40 mg/dl in men, <50 mg/dl in women or specific treatment for this lipid abnormality, elevated blood pressure (systolic ≥ 130 or diastolic ≥ 85 mmHg) or treatment for previously diagnosed hypertension, and previously diagnosed T2DM [16].

The diagnosis of CKD was established following the Kidney Disease Improving Global Outcomes (KDIGO) criteria, which consider eGFR and albuminuria levels, characterized by an eGFR below 60 ml/min/1.73m^2^. Microalbuminuria was identified by an UACR ranging from 30 to 300 mg/g, while macroalbuminuria was diagnosed in patients with UACR values surpassing 300 mg/g [1].

The CKD risk of progression category was set following the KDIGO and ADA criteria and stratified accordingly into low risk or Green, moderately increased risk or Yellow, high risk or Orange, and very high risk or Red, as follows: low, including an eGFR ≥90 ml/min/1.73m^2^ and UACR<30 mg/g or eGFR between 60 and 89 ml/min/1.73m^2^ and UACR between 30 and 299 mg/g; moderately increased, for an eGFR≥90 ml/min/1.73m^2^ and UACR between 30 and 299 mg/g or eGFR between 60 and 89 ml/min/1.73m^2^ and UACR between 30 and 299 mg/g or eGFR between 45 and 59 ml/min/1.73m^2^ and UACR <30 mg/g; high risk, including an eGFR≥90 ml/min/1.73m^2^ and UACR≥ 300 mg/g or eGFR between 60 and 89 ml/min/1.73m^2^ and UACR≥ 300 mg/g or eGFR between 45 and 59 ml/min/1.73m^2^ and UACR between 30 and 299 mg/g or ≥300 mg/g or eGFR between 30 and 44 ml/min/1.73m^2^, irrespective of the level of albuminuria or eGFR between 15 and 29 ml/min/1.73m^2^ and UACR< 30 mg/g or between 30 and 299 mg/g; very high risk, with an eGFR between 15 and 29 ml/min/1.73m^2^ and UACR≥300 mg/g or eGFR<15 ml/min/1.73m^2^, irrespective of the level of albuminuria [1,2].

Eye fundus examination, as part of the ophthalmological assessment, was conducted to evaluate diabetic retinopathy, following the protocol outlined in The Early Treatment for Diabetic Retinopathy Study [17].

The ankle-brachial index test was performed to assess peripheral arterial disease, with a diagnosis determined by the ankle-to-brachial blood pressure ratio. A value below 0.9 indicated the presence of the disease [2].

Statistical analysis

Statistical analysis was conducted using IBM SPSS Statistics for Windows, Version 19 (Released 2010; IBM Corp., Armonk, New York, United States). Continuous variables with a normal distribution were expressed as mean ± SD (standard deviation), while non-normally distributed variables were presented as median ± IQR (interquartile range). Categorical variables were reported as absolute counts and percentages. Normality was assessed using the Kolmogorov-Smirnov test with Lilliefors correction for significance and the Shapiro-Wilk statistic. Group comparisons for quantitative variables were made using analysis of variance (ANOVA), while the χ2 test was used for categorical variables. Non-normally distributed variables were analyzed using the Kruskal- Wallis test. Binary logistic regression and multiple regression analysis were performed to identify factors associated with very high risk of progression to dialysis. Statistical significance was set at a 95% confidence level.

## Results

The prevalence of CKD was higher among the Roma patients, reaching 56.50% (n=203), in comparison with the Caucasian group (43.50%, n=156). The paraclinical examinations and anthropometric measurements are presented and classified according to ethnicity and the existence of CKD as shown in Table 1. The average age of the Roma patients with CKD is lower compared to Caucasians (55.53±10.56 versus 63.32±10.04 years). The same trend was observed regarding the evolution of diabetes, with Roma patients with CKD having a significantly lower median duration of the disease (5.00±8.80 versus 11.50±12.00 years). The glycemic parameters revealed not only a higher mean HbA1c among the Roma patients with CKD, but also a higher median FBG (10.04±2.46% versus 9.13±1.94%, and 233.00±146.00 mg/dl versus 229.50±125.25 mg/dl, respectively). The mean values of the TyG index and ABSI were also higher among the Roma participants. Concerning the renal profile, among patients with CKD, the average values of creatinine and uric acid were higher among the Roma patients, but with no differences regarding the median UACR level (133.07±0.0001 mg/g versus 133.07±173.79 mg/g). Variables with statistically significant differences (p<0.05) between Caucasians and Roma participants with CKD are age, duration of diabetes, height, weight, WC, HC, SBP, DBP, HbA1c, TC, HDL-c, TG, LDL-c, TyG index, ABSI, UACR, and ALT.

**Table 1:** Anthropometric measurements and laboratory parameters stratified by ethnicity and the presence of diabetic CKD WC (cm): Waist circumference, HC (cm): hip circumference, BMI (kg/m^2^): body mass index,  SBP (mmHg): systolic blood pressure, DBP (mmHg): diastolic blood pressure, HbA1c (%): glycated hemoglobin,  FPG (mg/dl): fasting plasmatic glycemia, TC (mg/dl): total cholesterol, HDL-c (mg/dl): high-density lipoprotein-cholesterol, LDL-c (mg/dl): low-density lipoprotein-cholesterol, TG (mg/dl):  triglycerides,  TyG index:  triglyceride-glucose index, ABSI: A Body Shape Index, eGFR (ml/min/1.73m^2^): estimated glomerular filtration rate, UACR (mg/g): urinary albumin to creatinine ratio,  AST (UI/l): aspartate aminotransferase, ALT (UI/l): alanine aminotransferase, GGT (UI/l): gamma-glutamyl transferase, CKD: chronic kidney disease The data has been represented as mean± SD (standard deviation) and median± IQR (marked with "*", IQR- interquartile range). The statistical significance was considered at p-value<0.05. ** between Caucasians and Roma patients with CKD *** between Caucasians and Roma patients without CKD p-values that are statistically significant are written in bold

Parameters	Caucasian patients		Roma patients		p-value**	p-value***	
	With CKD (n=156)	Without CKD (n=177)	With CKD (n=203)	Without CKD (n=199)			
Age (years)	63.32±10.04	61.75±10.48	55.53±10.56	58.90±9.66	0.006	<0.001	
Duration of diabetes (years)	11.50±12.00*	12.00±10.50*	5.00±8.80*	7.00±10.00*	<0.001	<0.001	
Height (cm)	166.99±9.18	167.07±10.12	164.75±8.65	164.27±8.59	0.004	0.019	
Weight (kg)	85.59±17.66	85.58±17.77	92.11±19.33	89.82±17.31	0.028	0.002	
WC (cm)	105.67±12.19	105.03±11.37	110.17±11.17	110.39±10.00	<0.001	<0.001	
HC (cm)	104.83±16.94	104.94±11.21	111.07±13.41	110.85±13.51	0.002	0.008	
BMI (kg/m^2^)	32.61±6.60	31.57±5.41	33.19±6.15	33.48±5.90	0.001	0.391	
SBP (mmHg)	136.47±22.52	135.07±18.63	145.63±22.91	146.86±23.16	<0.001	0.002	
DBP (mmHg)	78.71±12.15	80.10±11.14	84.92±12.50	84.07±13.43	0.012	<0.001	
HbA1c (%)	9.13±1.94	9.03±2.32	10.04±2.46	9.99±2.49	<0.001	<0.001	
FPG (mg/dl)	229.50±125.25*	210.00±119.50*	233.00±146.00*	226.50±158.75*	0.011	0.218	
TC (mg/dl)	187.08±59.94	199.07±70.78	221.60±62.84	214.01±57.78	0.032	<0.001	
HDL-c (mg/dl)	47.66±12.17	50.00±14.97	45.29±9.45	45.56±8.54	<0.001	0.039	
TG (mg/dl)	153.50±130.90*	150.00±117.01*	214.54±140.00*	214.54±93.00*	0.003	<0.001	
LDL-c (mg/dl)	94.36±42.29	109.66±38.63	113.65±38.38	114.51±36.61	0.323	<0.001	
TyG index	9.81±0.74	9.68±0.81	10.10±0.68	10.05±0.61	<0.001	<0.001	
ABSI	0.81±0.10	0.82±0.09	0.84±0.087	0.83±0.09	0.099	0.003	
Creatinine (mg/dl)	1.06±0.43	0.88±0.29	1.01±0.37	1.04±0.49	<0.001	0.343	
eGFR (ml/min/1.73m^2^)	66.96±21.06	92.86±17.70	63.05±19.16	97.74±15.60	0.005	0.067	
Urea (mg/dl)	49.27±22.45	40.07±16.48	44.13±17.59	43.78±17.52	0.104	0.067	
Uric acid (mg/dl)	6.05±2.11	5.92±1.85	6.62±2.65	5.83±2.04	0.823	0.248	
UACR (mg/g)	133.07±173.79*	21.52±70.05*	133.07±0.0001*	133.07±109.07*	<0.001	<0.001	
AST (UI/l)	20.00±11.96*	20.43±11.08*	23.00±15.75*	24.00±15.00*	0.352	0.060	
ALT (UI/l)	23.00±19.21*	25.00±16.44*	27.00±23.25*	29.00±26.00*	0.035	0.006	
GGT (UI/l)	33.45±66.26*	30.77±49.83*	40.00±38.25*	46.00±32.00*	0.643	0.053	

Regarding the stage of CKD, a greater proportion of the Roma patients included had an eGFR between 45 and 59 ml/min/1.73m^2^ or between 30 and 44 ml/min/1.73m^2^ compared to the Caucasians (13.90% versus 11.40%, and 6.20% versus 5.40%, respectively). There were slight differences concerning the proportion of patients with an eGFR between 15 and 29 ml/min/1.73m^2^ (2.70% among the Roma and 2.40% among Caucasians). There were no patients with an eGFR below 15 ml/min/1.73m^2^.

As regards the level of albuminuria, a significant number of the Roma patients had an UACR between 30 and 300 mg/g (78.40% versus 48.90%, p<0.001), while more Caucasians had an UACR level above 300 mg/g (8.70% versus 1.70%, p<0.001) (Table 2).

**Table 2:** Proportion of the patients according to the eGFR and UACR level stratified by their ethnicity eGFR (ml/min/1.73m^2^): Estimated glomerular filtration rate, UACR (mg/g): urinary albumin to creatinine ratio The statistical significance was considered at p-value<0.05. p-values that are statistically significant are written in bold.

	Caucasian patients	Roma patients	Total	p-value
eGFR≥90 ml/min/1.73m^2^	38.10% (n=127)	37.30% (n=150)	37.70% (n=277)	0.819
eGFR 60-89 ml/min/1.73m^2^	43.20% (n=144)	39.60% (n=159)	41.20% (n=303)	0.328
eGFR 45-59 ml/min/1.73m^2^	11.40% (n=38)	13.90% (n=56)	12.80% (n=94)	0.320
eGFR 30-44 ml/min/1.73m^2^	5.40% (n=18)	6.20% (n=25)	5.90% (n=43)	0.753
eGFR 15-29 ml/min/1.73m^2^	2.40% (n=8)	2.70% (n=11)	2.60% (n=19)	0.820
eGFR<15 ml/min/1.73m^2^	0% (n=0)	0% (n=0)	0% (n=0)	
UACR <30 mg/g	42.30% (n=141)	20.10% (n=81)	30.20% (n=222)	<0.001
UACR 30-300 mg/g	48.90% (n=163)	78.40% (n=315)	65.00% (n=478)	<0.001
UACR >300 mg/g	8.70% (n=29)	1.70% (n=7)	4.90% (n=36)	<0.001

Taking into consideration the natural progression of CKD, the anthropometric measurements and laboratory parameters stratified by ethnicity and the CKD risk of progression category are found in Table 3 and Table 4. Roma patients in the very high risk of CKD progression category had a lower mean age, as well as a lower median duration of diabetes compared to Caucasians (56.37±10.79 versus 59.92±7.48 years, and 4.00±2.00 versus 10.00±10.30 years, respectively). Regarding the anthropometric parameters, Roma patients in the very high risk of CKD progression category had a more elevated mean WC and BMI compared to Caucasians; concerning the lipid profile, the mean TC and LDL-c were also higher among the first group. Moreover, patients in the very high risk of CKD progression category among both groups showed the highest level of insulin resistance, measured by the TyG index, with a mean value of 10.13±0.60 among the Roma participants, and 10.09±0.82 among Caucasians.

**Table 3:** Anthropometric measurements and laboratory parameters in Caucasian patients stratified by CKD risk of progression category WC (cm): Waist circumference, HC (cm): hip circumference, BMI (kg/m^2^): body mass index,  SBP (mmHg): systolic blood pressure, DBP (mmHg): diastolic blood pressure, HbA1c (%): glycated hemoglobin,  FPG (mg/dl): fasting plasmatic glycemia, TC (mg/dl): total cholesterol, HDL-c (mg/dl): high-density lipoprotein-cholesterol, LDL-c (mg/dl): low-density lipoprotein-cholesterol, TG (mg/dl): triglycerides,  TyG index: triglyceride-glucose index, ABSI: A Body Shape Index, eGFR (ml/min/1.73m^2^): estimated glomerular filtration rate, UACR (mg/g): urinary albumin to creatinine ratio,  AST (UI/l): aspartate aminotransferase, ALT (UI/l): alanine aminotransferase, GGT (UI/l): gamma-glutamyl transferase, CKD: chronic kidney disease The data has been represented as mean±SD (standard deviation) and median±IQR (marked with "*", IQR- interquartile range). The statistical significance was considered at p-value<0.05. p-values that are statistically significant are written in bold.

CKD risk of progression category	Caucasian patients				p-value
	Low	Moderately increased	High	Very high	
Age (years)	61.90±10.81	62.85±9.99	64.09±11.22	59.92± 7.48	0.365
Duration of diabetes (years)	12.00±10.00*	12.78±11.00*	10.50±10.80*	10.00±10.30*	0.853
Height (cm)	166.58±10.84	167.66±9.59	167.89±7.02	163.54±8.25	0.238
Weight (kg)	85.36±17.84	86.40±17.31	85.84±20.27	80.96±14.43	0.576
WC (cm)	105.99±11.22	105.07±10.11	107.05±15.74	100.82±14.74	0.126
HC (cm)	105.74±11.95	105.63±10.17	103.04±23.95	98.57±9.58	0.533
BMI (kg/m^2^)	30.64±4.56	32.80±6.28	31.61±6.63	34.64±7.53	0.004
SBP (mmHg)	133.66±16.86	135.45±22.44	138.94±21.51	141.67±26.31	0.455
DBP (mmHg)	79.10±10.57	79.79±12.49	80.15±12.33	77.50±11.38	0.899
HbA1c (%)	8.93±2.24	9.11±2.17	9.34±2.02	8.99±1.92	0.763
FPG (mg/dl)	215.00±125.00*	218.50±125.75*	250.00±126.75*	231.50±141.00*	0.174
TC (mg/dl)	198.13±66.24	191.43±66.98	196.04±68.29	179.92±56.38	0.633
HDL-c (mg/dl)	47.99±14.22	50.66±14.47	44.58±10.60	49.83±10.04	0.058
TG (mg/dl)	155.12±105.51*	150.41±133.69*	209.00±175.94*	134.10±74.75*	0.022
LDL-c (mg/dl)	112.82±38.10	97.92±42.74	95.37±38.97	82.54±42.83	0.031
TyG index	9.65±0.79	9.73±0.77	10.09±0.82	9.57±0.53	0.011
ABSI	0.84±0.08	0.80±0.10	0.83±0.09	0.75±0.12	<0.001
Creatinine (mg/dl)	0.84±0.27	1.00±0.38	1.18±0.51	0.91±0.22	<0.001
eGFR (ml/min/1.73m^2^)	88.66±19.01	85.44±18.57	68.72±21.75	35.84±8.43	<0.001
Urea (mg/dl)	38.85±16.60	47.25±22.62	49.72±20.13	46.81±13.93	0.020
Uric acid (mg/dl)	5.69±1.84	6.44±2.34	5.84±1.50	5.42±1.31	0.283
UACR (mg/g)	11.00±15.79*	133.07±79.83*	431.56±827.23*	133.07±299.90*	<0.001
AST (UI/l)	20.40±13.32*	20.00±10.00*	19.46±7.60*	24.00±23.95*	0.394
ALT (UI/l)	24.00±18.96*	24.00±16.00*	22.00±15.02*	31.00±25.75*	0.480
GGT (UI/l)	34.27±79.34*	41.50±65.57*	24.27±15.46*	282.60±556.19*	<0.001

**Table 4:** Anthropometric measurements and laboratory parameters in Roma patients stratified by CKD risk of progression category WC (cm): Waist circumference, HC (cm): hip circumference, BMI (kg/m2): body mass index,  SBP (mmHg): systolic blood pressure, DBP (mmHg): diastolic blood pressure, HbA1c (%): glycated hemoglobin,  FPG (mg/dl): fasting plasmatic glycemia, TC (mg/dl): total cholesterol, HDL-c (mg/dl): high-density lipoprotein-cholesterol, LDL-c (mg/dl): low-density lipoprotein-cholesterol, TG (mg/dl):  triglycerides,  TyG index:  triglyceride-glucose index, ABSI: A Body Shape Index, eGFR (ml/min/1.73m2): estimated glomerular filtration rate, UACR (mg/g): urinary albumin to creatinine ratio,  AST (UI/l): aspartate aminotransferase, ALT (UI/l): alanine aminotransferase, GGT (UI/l): gamma-glutamyl transferase, CKD: chronic kidney disease The data has been represented as mean±SD (standard deviation) and median±IQR (marked with "*", IQR- interquartile range). The statistical significance was considered at p-value<0.05. p-values that are statistically significant are written in bold.

CKD risk of progression category	Roma patients				p-value
	Low	Moderately increased	High	Very high	
Age (years)	57.23±8.39	57.86±10.25	54.74±11.59	56.37±10.79	0.248
Duration of diabetes (years)	4.00± 9.00*	7.00±10.00*	5.00±10.00*	4.00± 2.00*	0.618
Height (cm)	165.34±7.78	164.00±8.62	165.17±9.15	165.83±8.02	0.811
Weight (kg)	89.82±16.33	90.42±18.73	90.17±17.47	96.74±16.52	0.460
WC (cm)	110.27±10.71	110.49±10.62	109.40±9.13	112.97±11.36	0.190
HC (cm)	110.34±13.80	111.21±14.04	107.96±8.22	117.25±13.57	0.250
BMI (kg/m^2^)	33.02±5.51	33.43±6.12	31.84±5.17	35.66±6.42	0.053
SBP (mmHg)	145.85±21.08	146.59±23.72	144.84±21.24	147.25±26.63	0.964
DBP (mmHg)	85.27±14.24	84.00±12.53	85.20±12.96	85.65±14.52	0.849
HbA1c (%)	9.47±2.29	10.11±2.50	10.09±2.57	10.12±2.43	0.518
FPG (mg/dl)	210.00±125.50*	230.00±165.50*	239.00±90.50*	254.00±198.50*	0.104
TC (mg/dl)	217.21±65.74	217.77±59.66	212.38±53.98	231.24±67.41	0.657
HDL-c (mg/dl)	44.69±9.10	45.47±8.65	45.40±9.82	47.05±10.76	0.735
TG (mg/dl)	214.54±121.00*	214.54±113.00*	214.54±131.50*	170.00±231.00*	0.641
LDL-c (mg/dl)	116.28±38.06	114.14±37.14	110.07±37.02	116.03±41.93	0.888
TyG index	9.93±0.58	10.10±0.62	10.13±0.60	10.02±0.99	0.269
ABSI	0.84±0.08	0.84±0.09	0.85±0.09	0.81± 0.07	0.306
Creatinine (mg/dl)	0.87±0.23	1.06±0.45	1.12±0.50	0.92± 0.23	0.012
eGFR (ml/min/1.73m^2^)	89.06±16.63	88.25±18.95	54.66±17.96	33.97±9.94	<0.001
Urea (mg/dl)	42.25±16.26	44.01±18.42	46.00±16.47	43.90±17.83	0.799
Uric acid (mg/dl)	6.01±2.51	6.03±2.23	5.89±1.82	8.42± 3.12	0.086
UACR (mg/g)	20.00±11.00*	133.07±0.0001*	133.07±0.0001*	133.07±0.0001	<0.001
AST (UI/l)	24.00±15.75*	24.00±16.00*	24.00±14.50*	22.00±17.00*	0.917
ALT (UI/l)	35.00±24.00*	29.00±27.00*	32.50±23.50*	28.00±31.50*	0.610
GGT (UI/l)	40.00±26.00*	45.00±32.00*	56.00±36.00*	48.00±44.00*	0.935

Concerning the associated diseases, Roma patients with CKD had a higher prevalence of cardiovascular disease compared to Caucasians, including myocardial infarction, stroke, stable angina, and heart failure. However, peripheral artery disease was more present among the Caucasian group (54.30% versus 40.50%). Cardiovascular risk factors, such as hypertension, obesity, and dyslipidemia, in patients with CKD, were also more prevalent among the Roma. There were little differences with regard to the metabolic syndrome, a slightly higher percentage being observed among the Roma patients (50.00% versus 47.20%) (Table 5).

**Table 5:** Prevalence of comorbidities stratified by ethnicity and the presence of CKD The statistical significance was considered at p-value<0.05. *between Caucasians and Roma patients with CKD ** between Caucasians and Roma patients without CKD CKD: Chronic kidney disease p-values that are statistically significant are written in bold

Parameters	Caucasian patients		Roma patients		p-value*	p-value**
	With CKD (n=156)	Without CKD (n=177)	With CKD (n=203)	Without CKD (n=199)		
Myocardial infarction	58.50% (n=24)	41.50% (n=17)	46.30% (n=25)	53.70% (n=29)	0.158	0.443
Stroke	57.90% (n=11)	42.10% (n=8)	51.20% (n=21)	48.80% (n=20)	0.049	0.351
Stable angina	43.20% (n=19)	56.80% (n=25)	50.40% (n=61)	49.60% (n=60)	<0.001	<0.001
Peripheral artery disease	54.30% (n=38)	45.70% (n=32)	40.50% (n=17)	59.50% (n=25)	0.151	<0.001
Lower limb amputation	41.70% (n=5)	58.30% (n=7)	43.80% (n=7)	56.30% (n=9)	0.805	1.00
Heart failure	61.10% (n=11)	38.90% (n=7)	56.60% (n=43)	43.40% (n=33)	<0.001	<0.001
Hypertension	47.40% (n=130)	52.60% (n=144)	48.10% (n=139)	51.90% (n=150)	0.001	0.171
Obesity	45.80% (n=98)	54.20% (n=116)	47.60% (n=141)	52.40% (n=155)	0.008	0.215
Dyslipidemia	42.20% (n=147)	46.60% (n=172)	57.80% (n=201)	53.40% (n=197)	0.012	0.261
Hepatic steatosis	51.30% (n=40)	48.70% (n=38)	45.50% (n=95)	54.50% (n=114)	0.330	0.029
Metabolic syndrome	47.20% (n=142)	52.80% (n=159)	50.00% (n=196)	50.00% (n=196)	0.062	<0.001

As regards the prevalence of the other diabetic microvascular complications, a greater number of the Roma patients with CKD had peripheral polyneuropathy and orthostatic hypotension (136 versus 125 patients, p=0.011, and 34 versus 18 patients, p=0.056), while retinopathy was significantly more prevalent among the Caucasian group (68 versus 59 patients, p=0.023) (Table 6). 

**Table 6:** Prevalence of diabetic complications stratified by ethnicity and the presence of CKD The statistical significance was considered at a p-value<0.05. *between Caucasians and Roma patients with CKD ** between Caucasians and Roma patients without CKD CKD: Chronic kidney disease p-values that are statistically significant are written in bold.

Parameters	Caucasian patients		Roma patients		p-value*	p-value**
	With CKD (n=156)	Without CKD (n=177)	With CKD (n=203)	Without CKD (n=199)		
Diabetic peripheral polyneuropathy	80.10% (n=125)	77.40% (n=137)	46.70% (n=136)	53.30% (n=155)	0.011	1.00
Orthostatic hypotension	66.70% (n=18)	33.30% (n=9)	63.00% (n=34)	37.00% (n=20)	0.056	0.043
Diabetic retinopathy	55.30% (n=68)	44.70% (n=55)	47.20% (n=59)	52.80% (n=66)	0.387	0.023

Factors contributing to the progression of CKD

We conducted univariate analysis on age, sex, weight, WC, BMI, SBP, triglyceride level, ABSI, and TyG index to evaluate if these factors are contributors to the progression of CKD. The variables significantly associated with a very high risk of progression of CKD were included in a binary logistic regression analysis.

Multivariate analysis of factors associated with very high risk of CKD progression category

On multivariate analysis, among the study group, weight, WC, BMI, and ABSI were associated with a very high risk of progression of CKD (Table 7). In Caucasian patients, it was demonstrated that weight, WC, BMI, TG, and ABSI have contributed to the very high risk of progression of CKD, while among the Roma patients, no association was found (Table 8).

**Table 7:** Factors associated with very high risk of progression of CKD among the study patients WC (cm): Waist circumference, BMI (kg/m^2^): body mass index, TG (mg/dl): triglycerides, ABSI: A Body Shape Index, TyG index: triglyceride-glucose index, B: standard beta coefficient, SE: standard error. The statistical significance was considered at a p-value<0.05. CKD: Chronic kidney disease p-values that are statistically significant are written in bold.

Variables	B	SE	p-value	
				Age (years)	-0.033	0.028	0.239	
Weight (kg)	-0.161	0.072	0.025	
WC (cm)	0.521	0.225	0.021	
BMI (kg/m^2^)	-0.682	0.325	0.036	
TG (mg/dl)	-0.004	0.004	0.280	
ABSI	-69.16	28.664	0.016	
TyG index	0.314	0.579	0.587	

**Table 8:** Factors associated with very high risk of progression of CKD stratified by ethnicity WC (cm): Waist circumference, BMI (kg/m^2^): body mass index, TG (mg/dl): triglycerides, ABSI: A Body Shape Index, TyG index: triglyceride-glucose index, B: standard beta coefficient, SE: standard error, CI: confidence interval. The statistical significance was considered at a p-value<0.05. CKD: Chronic kidney disease p-values that are statistically significant are written in bold.

Variables	B	SE	p-value	95% CI	
				Lower	Upper
Caucasian patients					
Age (years)	-0.003	0.043	0.942	0.916	1.084
Weight (kg)	-0.623	0.254	0.039	0.360	0.975
WC (cm)	1.506	0.753	0.046	1.030	19.744
BMI (kg/m^2^)	-2.183	1.086	0.044	0.013	0.946
TG (mg/dl)	-0.026	0.013	0.041	0.951	0.999
ABSI	-183.659	89.87	0.041	0.0001	0.001
TyG index	1.669	1.130	0.140	0.580	48.641
Roma patients					
Age (years)	-0.090	0.049	0.067	0.831	1.006
Weight (kg)	-0.132	0.101	0.192	0.719	1.068
WC (cm)	0.520	0.324	0.109	0.891	3.176
BMI (kg/m^2^)	-0.682	0.453	0.133	0.208	1.230
TG (mg/dl)	-0.002	0.005	0.729	0.989	1.007
ABSI	-62.792	43.940	0.153	0.0001	1.355
TyG index	0.120	0.783	0.879	0.243	5.232

## Discussion

Current medical knowledge recognizes a prevalence of CKD in patients with diabetes between 20 and 40% [2]. Our study identified a prevalence of 43.50% among the Caucasian patients; however, among the Roma group, a higher percentage of patients had CKD (56.50%).

In the PREDATORR (PREvalence of DiAbeTes mellitus, prediabetes, overweight, Obesity, dyslipidemia, hyperuricemia and chronic kidney disease in Romania) study, one representative paper in Romania, the prevalence of CKD was 9.08%, although only 11.6% of the patients included had diabetes. Moreover, regarding the paraclinical and anthropometric measurements, BMI, WC, FPG, HbA1c, TG, uric acid, SBP, and insulin resistance measured with the HOMA-IR index were higher among the participants with CKD. Apart from this, 0.52% of the participants had an eGFR below 29 ml/min/1.73m^2^ and 0.48% had macroalbuminuria [18]. Accordingly, in our paper, among both groups of patients, markers such as BMI, WC, FPG, HbA1c, uric acid, SBP, and TyG index were also higher in patients with CKD. Regarding the proportion of patients according to the eGFR and UACR, our results showed higher percentages, with 2.60% of the participants having an eGFR between 15 and 29 ml/min/1.73m^2^, while 4.90% of the patients had macroalbuminuria; however, there were no patients with an eGFR below 15 ml/min/1.73m^2^.

Ethnic differences in the occurrence of CKD have been documented in various parts of the world, with individuals from the Roma population being more often diagnosed, although existing data refer mostly to ESRD. Roma patients from Slovakia have a 34% higher risk of being diagnosed with ESRD [7]. It was also reported that the Roma population has a 2- to 3-fold increased risk of ESRD compared with the general population [19]. Concerning the causes of ESRD, diabetic nephropathy was more frequent among the Roma population (24.10%) compared to the general population (19.50%) [20]. Moreover, another study from the medical literature regarding dialyzed Roma patients in Slovakia identified that the prevalence of diabetic nephropathy accounts for 34% of the causes [21].

The prevalence of CKD is known to increase with age [22,23]. In our paper, the mean age of the Roma patients with CKD was lower (55.53±10.56 years), while among the Caucasians, it was 63.32±10.04 years, nonetheless, the prevalence of CKD was higher among the first group (56.50% versus 43.50%).

Comparing different parameters among patients with CKD with those without CKD, Chen et al identified that there were no differences regarding age, SBP, BMI, eGFR, prevalence of cardiovascular disease, and hypertension [24]. In our study, however, there were differences, not only regarding the presence of CKD but also between the two ethnic groups. Roma patients, regardless of the diagnosis of CKD, had a lower mean age, and higher average value of SBP and BMI compared to Caucasians. Nevertheless, the mean eGFR was lower among the Roma group with CKD, but higher among those without CKD, compared to the corresponding group. Cardiovascular disease, represented by myocardial infarction, stroke, stable angina, peripheral artery disease, and heart failure was more prevalent among the Roma, irrespective of the presence of CKD, while regarding hypertension, more Roma patients had this condition compared to Caucasians.

In patients with type 2 diabetes, cardiovascular disease remains the leading cause of morbidity and mortality, with many systematic reviews showing that these patients have about twice the risk of developing cardiovascular disease compared to those without diabetes. In the CAPTURE study, a multinational, cross-sectional study that included 13 countries, over one-third of the patients included (reaching 36.50%) had established cardiovascular disease. Moreover, a higher proportion of participants from this group had hypertension (82.90% versus 62.7%), an eGFR lower than 59 mL/min/1.73 m^2^ (30.70% versus 15.40%), microalbuminuria (31.80% versus 20.60%) and macroalbuminuria (10.80% versus 6.80%), in comparison with the non-cardiovascular disease group. The prevalence of nephropathy was higher among patients with cardiovascular disease (29.20% versus 17.00%), as well as the other microvascular complications (retinopathy 24.30% versus 15.80%, and neuropathy 29.00% versus 19.20%) [25]. Data from our study also suggest a significant prevalence of cardiovascular disease, irrespective of their chronic kidney disease status or ethnicity, although Roma patients with CKD had a higher prevalence of cardiovascular disease compared to Caucasians, and microvascular complications.

Furthermore, cardiovascular diseases, hypertension, and dyslipidemia are known to be more prevalent among patients with CKD [26], with previous studies having extensively demonstrated the association between CKD and these diseases [27]. The prevalence of hypertension ranges from 60% to 90%, depending on the stage and cause of CKD [28], while the prevalence of dyslipidemia was shown to be around 35.80% in men and 54.70% in women with CKD. A paper from Iran showed that 33.50% of the patients with CKD had hypertension, 3.30% had myocardial infarction, and 2.10% had a stroke, twice as much as the patients without CKD [27]. Our results are consistent with these findings, these comorbidities being more prevalent among both groups with CKD.

Concerning insulin resistance, Ádány et al. examined the TyG index in a sample of the Roma population from Hungary and found no significant differences when compared to a corresponding group from the general Hungarian population, with both groups having an average value of 4.88 [29]. In contrast, our findings suggested a higher mean TyG index value in the Roma group with CKD compared to Caucasians (10.09±0.82 versus 9.65±0.79), with a lower value among those without CKD (9.57±0.53 versus 9.73±0.77, respectively).

The finding in our paper that weight, WC, BMI, TG, and ABSI contribute to the very high risk of CKD progression in Caucasian patients, while no such association was observed among Roma patients, warrants further exploration. According to Chen et al., generalized obesity is associated with an increased risk of diabetic microvascular complications in patients with type 2 diabetes [4]. One potential explanation for this discrepancy could be the different "phenotypes" of diabetic CKD between the two groups, such as physiological responses and biochemical markers, that may differ between ethnic groups due to a combination of genetic, environmental, and lifestyle factors [20].

Limitations

In addressing the limitations of our research, it is important to note that participant recruitment was limited to a single tertiary care hospital during the COVID-19 pandemic. This may not accurately reflect the broader Roma and non-Roma populations with diabetes, although our sample size is reasonable. Our findings are consistent with larger studies comparing the general population to ethnic minorities. However, there is a notable lack of data on Roma patients. One other limitation that we acknowledge is the absence of data on the eGFR slope, which is an accepted surrogate outcome for the progression of CKD. Without this information, it is challenging to accurately assess the rate of CKD progression among participants. Additionally, the study lacks hard clinical outcomes such as the need for dialysis or transplant, and mortality data, which are critical for evaluating the long-term impact of CKD and the effectiveness of interventions. Therefore, we emphasize the need for further research in this area, with a more detailed analysis to identify the specific genetic and environmental factors contributing to the risk progression of CKD in the Roma population. Conducting a longitudinal study to explore cause-and-effect relationships between the observed factors and health outcomes, in collaboration with community leaders and healthcare professionals from the Roma community, could greatly contribute to improving cardiovascular health outcomes and reducing ethnic disparities in healthcare for the Roma population.

## Conclusions

Taking the above into consideration, our findings suggested a prevalence of CKD of 43.50% among the Caucasian patients and 56.50% among the Roma group with T2DM. There were observed differences regarding various paraclinical parameters between these ethnic groups. Additionally, the Roma population exhibited a higher prevalence of associated diseases and cardiovascular risk factors. Among the study group, weight, WC, BMI, and ABSI were associated with a very high risk of progression of CKD. In Caucasian patients, it was demonstrated that weight, WC, BMI, TG, and ABSI have contributed to the very high risk of progression of CKD, while among the Roma patients, no association was found. These findings highlight the need for further investigation of additional risk factors, such as genetics, limited access to health education, and appropriate treatments that could synergistically accelerate the progression of CKD.
